# Identification of factors affecting fattening efficiency of commercial pig herds and analysis of their impact at different performance levels

**DOI:** 10.1038/s41598-024-70900-x

**Published:** 2024-08-29

**Authors:** Ran Guan, Zhiwei Zheng, Hai Yu, Lili Wu, He Huang, Ping Jiang, Xiaowen Li

**Affiliations:** 1 Shandong New Hope Liuhe Group Co., Ltd., No. 592-26, Jiushui East Road, Laoshan District, Qingdao, 266000 Shandong China; 2 Key Laboratory of Animal Disease Diagnostics and Immunology, Ministry of Agriculture, MOE International Joint Collaborative Research Laboratory for Animal Health & Food Safety, College of Veterinary Medicine, Nanjing Agricultural University, Nanjing, 210095 Jiangsu China; 3 College of Animal Science, Xichang University, Xichang, 615012 China

**Keywords:** Risk factors, Animal behaviour, Animal physiology

## Abstract

Improving fattening efficiency is an important goal of breeding commercial pigs, especially for the large-scale pig farms. Fattening efficiency index (FEI) can be used to evaluate the fattening efficiency. The aim of this study was to identify the factors affecting the fattening efficiency of commercial pigs in large-scale pig farms and further study the impact of these factors on the production performance of commercial pig batches at different production levels. The data of 9,570 batches was mainly consisted of four parts (farm facilities, general information of piglets, production performance of nursery pigs and finishing pigs). A total of 28 variables were evaluated by the multi-variable linear regression models. The differences in production factors significantly correlated with FEI at piglets-finishing stage were compared among high-performing (HP), moderate-performing (MP), and low-performing (LP) batches of commercial pigs during the nursery and finishing stage. Among the 28 variables, 18 were significantly correlated with fattening efficiency (*P* < 0.05), including 11 continuous variables and seven discrete variables. The significant differences among the 11 consecutive variables in the HP, MP, and LP batches of commercial pigs mostly persisted from the piglets-nursery stage to the growing-finishing stage, ultimately affecting the FEI at piglets-finishing stage. For the seven significant discrete variables, the HP batches had a lower proportions in owned source of piglets, number of the purchasing piglets in spring and winter, number of batches in the East and North regions and five-way crossbred pigs, while a higher proportions in the use of closed circuit television video (CCTV) and wastes treatment system. The fattening efficiency of commercial pigs in large-scale pig farms was comprehensively affected by farm facilities, piglets, and production performance at nursery and finishing stage. The low fattening efficiency may have started at the end of nursery stage.

## Introduction

With the impact of the epidemic of African swine fever (ASF) and government policies, China's pig farming industry is gradually shifting to large-scale and centralized development, and the market share occupied by the top enterprises is increasing^1^. Under the economies of scale, production efficiency has become particularly important^2^. Unsuitable feeding environments and the consumptive diseases will result in slow growth rates and reduced feed efficiency, leading to additional production costs^3^. This may be attributed to excessive consumption of body substances, resulting in negative energy balance. Due to the incomplete development of digestive and immune systems, nursery pigs are more prone to developing diseases than growing-finishing pigs^4^. As pigs grow, the management conditions for growing-finishing pigs become more complex, including increased herd density, aging facilities, accumulation of environmental pathogens, and adjustments to the nutritional content in feed^5,6^. Keeping the comfort of pigs is crucial to ensuring good production performance and preventing a decline in the efficiency of body energy utilization^7^.

The fattening efficiency (FE) of commercial pigs is of pivotal importance for the profitability and sustainability of large-scale pig farms. Previous studies have concentrated on individual factors, such as genetic improvement, nutritional optimisation and disease management. The research indicated that genetic selection can improve growth rate and feed conversion ratio (FCR); however, it often overlooked the interaction with environmental factors^8^. Similarly, nutritional studies emphasized the significance of balanced diets and amino acid supplementation in enhancing FE, but frequently neglect to consider the impact of farm infrastructure and management practices^9^. Although disease management strategies have been studied with regard to their role in reducing mortality and enhancing growth performance, they typically fail to consider the synergistic effects of other production variables^10^.

Nevertheless, a notable lacuna persists in our understanding of the multifaceted factors that shape FE across diverse production phases. The existing literature frequently lacks an integrated approach that considers the combined effects of farm facilities, environmental conditions, and management practices on FE. The current lack of consideration for these factors hinders the development of comprehensive strategies that could enhance production efficiency. This study aims to address these limitations by identifying a broad range of factors affecting the FE of commercial pigs in large-scale farms. By evaluating the impact of these factors on production performance across diverse pig batches, this research aims to provide a more comprehensive understanding of FE determinants, which can inform the development of strategies to optimise pig growth outcomes and enhance farm efficiency.

## Methods

The study did not require approval from the Ethics Committee on Animal Use, as no animal were handled during the research. All production data were sourced from the internal data management system of a large-scale pig farming company (hereinafter referred to as “the company”). The researchers obtained authorization from both the company’s production management department and digital technology department.

### Farm description

This study was conducted in 2,574 farms who cooperated with the company and included 7,752,278 commercial pigs of 9,570 batches in 2022. The basic conditions of all pig farms were detailed in our previous studies^11^.These farms were from 24 provinces (autonomous regions or municipalities directly under the Central Government), located in the various regions of the country, namely, the East China (27.6%), North China (5.8%), South China (29.6%), Central China (11.8%), Northwest (9.4%), Northeast (3.8%) and Southwest (12.0%) regions. Piglets, veterinarians, and technical services are uniformly arranged by the company. Pigs were fed with the corresponding formula of standardized feed (10 kinds of feeds in the stages of nursery, growth, fattening) provided by the company's internal feed factory. All production data of these farms were uploaded to the internal data management system of the company.

### Data collection and manipulation

The researchers were authorized by the company's production management department and digital technology department to obtain the production data in this study. The batch data mainly consisted of four parts: farm facilities, general information of piglets, production performance of nursery pigs and finishing pigs (see Supplementary Table S1 online). They met the following conditions: (1) Complete data records; (2) Nonzero; (3) Age of piglets was up to 40 days; (4) Body weight (BW) of nursery pigs at 70 days was 15 to 35 kg; (5) Survival rate (SR) of nursery pigs at 70 days was greater than or equal to 80%; (6) FCR was 1 to 5. Therefore, a total of 9,570 batch data were included for the further analysis.

### Definitions

In previous research, we proposed a new production index for commercial pigs—fattening efficiency index (FEI)^11^. The FEI of different growth stages was calculated through corresponding production performance (Table 1). The formula of FEI was as follow:

$${\text{FEI}}\left( {\text{g}} \right) = {\text{SR}}\left( \% \right) \times {\text{ADG}}\left( {{\text{kg}}} \right) \times {1}000/{\text{FCR}}$$

**Table 1:** Reference growth performance of commercial pigs at different growth intervals.

Growth intervals	Body weight (kg)	Age (d)	Interval days	Feeds (kg)	ADG (kg)	ADFI (kg)	FCR	SR (%)	FEI (g)
Piglets-nursery	6–25	21–70	50	35.00	0.38	0.70	1.84	98.00	202.39
Growing-finishing	26–118	71–180	110	256.20	0.84	2.33	2.75	97.00	296.29
Piglets-finishing	6–118	21–180	160	291.20	0.70	1.82	2.60	95.00	255.77

*ADG* average daily gain, *ADFI* average daily feed intake, *FCR* feed conversion rate, *SR* survival rate, *FEI* fattening efficiency index.

### Statistics analysis

Discrete variables were divided according to: crossbred (three-way, five-way, two-way), gender (mixed, female, barrow), area of concrete grids (1/2, 1/3, 2/3), seasons of purchasing piglets (spring (March to May), summer (June to August), autumn (September to November), winter (December to February)), regions of pig farms (East China, North China, South China, Central China, Northwest, Northeast, Southwest). Enclosure, bio-safety zoning, environmental control system, automatic feeding system, closed circuit television video (CCTV), wastes treatment system, disinfection channel and bio-safety disposal was divided into “Yes” or “No”.

**Fig. 1:**
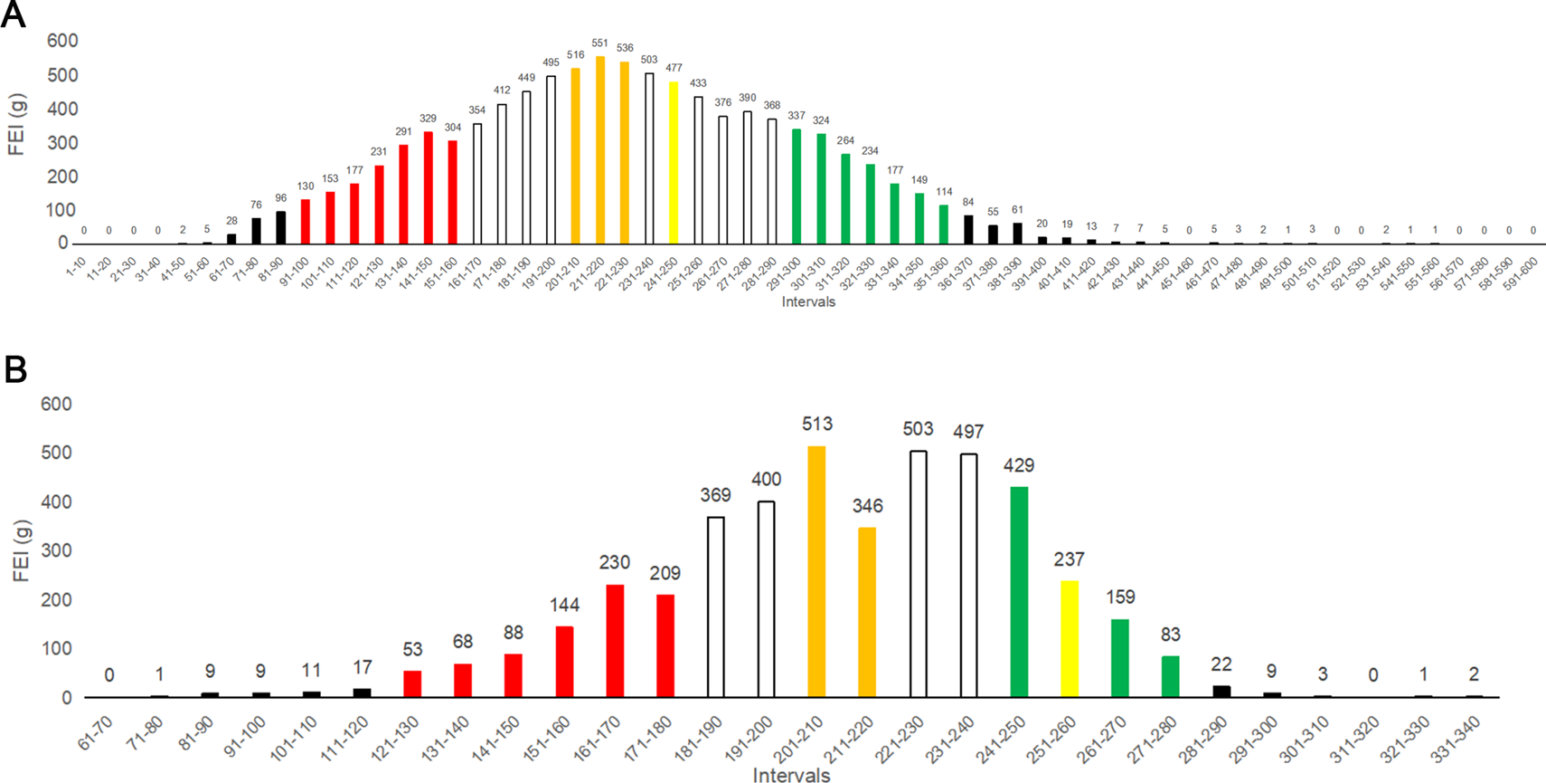
Distribution of fattening efficiency index at nursery stage (**A**) and finishing stage (**B**). Black represents the excluded intervals which is less than one thousandth. Red represents the intervals of low-performing batches. Orange represents the intervals of medium-performing batches. Green represents the intervals of high-performing batches. Yellow represents the reference interval of production performance at the corresponding stages. n (A) = 9,570 batches, n (B) = 4,412 batches.

The overall distribution of FEI at nursery stage (n = 9,570 batches) followed a normal distribution (Fig. 1A). After excluding intervals with less than 100 (1–90 intervals and 361–600 intervals), the continuous variables of remaining intervals were divided into three production performance groups: 1,599 high-performing (HP) batches (FEI: 291–360 g), 1,603 moderate-performing (MP) batches (FEI: 201–230 g), and 1,615 low-performing (LP) batches (FEI: 91–160 g). During the finishing stage (n = 4,412 batches), the overall distribution of FEI was close to a normal distribution (Fig. 1B). After excluding intervals with less than 50 (61–120 g and 281–340 g), the continuous variables of remaining intervals were divided into three production performance groups: 847 HP batches (FEI: 243–280 g), 865 MP batches (FEI: 204–223 g) and 841 LP batches (FEI: 121–182 g).

Descriptive statistics were conducted using WPS Office Excel for Windows, version 11.1.0.11830 (Kingsoft Office Corporation, Beijing, China). For continuous variables, the mean, standard deviation (SD), minimum, maximum and median were assessed. A multiple linear regression model was employed to evaluate the relationship between the FEI and a set of independent variables. The variables significantly correlated with FEI in univariate analysis were identified and utilized to refine the model through stepwise regression methods. Categorical variables were transformed into a dummy variable matrix for application in regression model calculations. The basic assumptions of the model, including linearity, homoscedasticity, and independence were tested. The multicollinearity was assessed using the variance inflation factor to ensure that the relationships between independent variables did not overly influence the model outcomes. The practical significance of the findings was conveyed through the presentation of *P* value and Cohen’s d effect sizes, along with their confidence intervals. All differential analyses were conducted using Graphpad Prism 8.4 (Graphpad Software, inc. San Diego, CA, USA). Dunnett's multiple comparisons test was applied to discern differences in production performance among HP, IP and LP batches. A threshold of *P* < 0.05 was set to denote statistical significance.

### Ethics approval and consent to participate

All data in this study came from the internal data management system, and the author had access rights. The study only involved statistical analysis of the data, without field investigation of pig farms.

## Results

Descriptive statistics of production items with 19 continuous variables of 9,570 commercial pig batches at piglets, nursery and finishing stage were shown in Table 2. Statistical analysis was conducted on 28 production items related to FEI during the piglets-finishing stages, of which 18 showed significant differences (*P* < 0.05), including 11 continuous variables and seven discrete variables (Table 3).

**Table 2:** Descriptive statistics on production performance of 9,570 commercial pig batches.

Items	Mean	SD	Min	Max	Median
Stock (n)	4,174.72	3,070.87	498	25,000	3,300
Number of cooperative batches (n)	2.97	2.65	1	25	2
Number of piglets sources (n)	2.33	1.68	1	12	2
Age of piglets (d)	25.87	3.78	18	40	25
BW of piglets (kg)	6.49	0.65	4.00	13.62	6.44
BW of nursery pigs at 70 days (kg)	20.94	2.50	15.01	32.99	21.15
SR of nursery pigs at 70 days (%)	95.90	3.69	80.01	100.00	97.11
Gain BW during the nursery stage (kg)	14.44	2.56	6.43	26.83	14.62
ADG during the nursery stage (kg)	0.33	0.06	0.16	0.64	0.33
ADFI during the nursery stage (kg)	0.48	0.10	0.23	1.29	0.46
FCR during the nursery stage (kg / kg)	1.48	0.34	1.01	3.89	1.41
FEI during the nursery stage (g)	226.84	71.69	43.45	559.96	224.57
Feeding days during the piglets-finishing stage (day)*	171.80	13.47	120	247	172
Number of finishing pigs during the piglets-finishing stage (n)*	2,772.46	1,686.20	101	9,136	2,336
BW of finishing pigs during the piglets-finishing stage (kg)*	118.19	10.90	65.51	162.56	119.95
ADG during the piglets-finishing stage (kg)*	0.65	0.05	0.38	0.81	0.65
SR during the piglets-finishing stage (%)*	89.12	6.32	65.15	100.00	90.58
FCR during the piglets-finishing stage (kg / kg)*	2.76	0.22	1.56	4.78	2.73
FEI during the piglets-finishing stage (g)*	211.28	35.67	65.85	489.01	213.93

*8894 in 9570 batches, excluding blank data (n = 9), feeding days < 120 d (n = 62), ADG ≤ 0 kg (n = 198), BW of finishing pigs < 65 kg (n = 335), SR < 65% (n = 55), and FCR < 1 and ≥ 5 (n = 17). *SD* standard deviation, *BW* body weight, *SR* survival rate, *ADG* average daily gain, *ADFI* average daily feed intake, *FCR* feed conversion rate, *FEI* fattening efficiency index.

**Table 3:** Coefficient analysis of 28 factors related to FEI at piglets-finishing stage.

Items		Partial regression coefficient	SEM of coefficient	Variance inflation factor	*P*-value
FEI	**Piglets-nursery (g)**	0.011164	0.000966	1.28	0.000
	**Growing-finishing (g)**	0.07878	0.00209	3.76	0.000
Piglets	**Number of piglets**	0.000405	0.000114	1.39	0.000
	**Number of piglets sources**	-0.2098	0.0462	1.47	0.000
	**Weaning BW (kg)**	0.365	0.124	1.61	0.003
	Age (d)	0.0420	0.0221	1.75	0.057
	**Sources of piglets**	N/A	N/A	N/A	0.000
	**Crossbred**	N/A	N/A	N/A	0.000
	Gender	N/A	N/A	N/A	0.248
	**Season for piglets**	N/A	N/A	N/A	0.000
Farm	Stock (n)	-0.000030	0.000022	1.27	0.175
	Number of cooperative batches (n)	0.0316	0.0286	1.17	0.271
	**Regions**	N/A	N/A	N/A	0.000
Piglets-nursery stage	**ADFI**	3.781	0.733	1.44	0.000
Piglets-finishing stage	**Feeding days (d)**	0.2471	0.0183	35.64	0.000
	**ADG (kg)**	367.24	4.93	29.94	0.000
	**BW of finishing pigs (kg)**	-0.5445	0.0332	84.58	0.000
	**SR (%)**	196.46	1.28	1.88	0.000
	**FCR**	-56.628	0.393	2.32	0.000
Basic facilities	**Area of concrete grids**	N/A	N/A	N/A	0.046
	Enclosure	N/A	N/A	N/A	0.856
	Bio-safety zoning	N/A	N/A	N/A	0.503
	Disinfection channel	N/A	N/A	N/A	0.941
	**Wastes treatment system**	N/A	N/A	N/A	0.000
	Automatic feeding system	N/A	N/A	N/A	0.679
	Environmental control system	N/A	N/A	N/A	0.857
	Bio-safety disposal	N/A	N/A	N/A	0.110
	**CCTV**	N/A	N/A	N/A	0.002

*P* < 0.05 means significant differences and *P* < 0.01 means extremely significant differences (bold characters). *N/A* not applicable, *SEM* standard error of mean, *FEI* fattening efficiency index, *BW* body weight, *ADFI* average daily feed intake, *ADG* average daily gain, *SR* survival rate, *FCR* feed conversion rate, *CCTV* closed circuit television video.

Table 4 showed the differences in production items (11 continuous variables in Table 3) significantly correlated with FEI at piglets-finishing stage between HP, MP, and LP commercial pig batches at the nursery stage and the finishing stage. Although the weaning BW of the three levels was similar, there were significant differences in FEI at the different stages, as well as the number of piglets sources and average daily feed intake (ADFI) at nursery stage and average daily gain (ADG) and SR at finishing stage.

**Table 4:** Analysis of differences of the production items of significant correlation with FEI at piglets-finishing stages in high, moderate, and low-performing batches of commercial pigs at nursery and finishing stage.

Stages	Production items	High-performing batches	Moderate-performing batches	Low-performing batches
		Mean ± SD	Mean ± SD	Mean ± SD
Piglets-nursery	Number of batches	1,599	1,603	1,615
	Number of piglets (n)	816.55 ± 612.65	801.49 ± 610.08	792.49 ± 648.82
	Weaning BW (kg)	6.47 ± 0.62	6.46 ± 0.60	6.50 ± 0.69
	Number of piglets sources (n)	2.44 ± 1.78^a^	2.30 ± 1.61^b^	2.32 ± 1.72^b^
	ADFI (kg)	19.44 ± 3.16^a^	21.22 ± 4.34^b^	21.97 ± 5.28^c^
	FEI (g)	318.93 ± 19.02^a^	216.17 ± 8.60^b^	132.12 ± 19.03^c^
Growing-finishing	Number of batches*	1,498	1,494	1,420
	FEI (g)	256.99 ± 117.48^a^	244.81 ± 71.20^b^	247.72 ± 79.61^b^
	Feeding days (d)	171.25 ± 13.09	171.35 ± 13.36	172.40 ± 13.55
	BW (kg)	119.07 ± 11.16^a^	118.02 ± 10.48^b^	117.10 ± 11.52^b^
	ADG (kg)	0.654 ± 0.055^a^	0.647 ± 0.053^b^	0.637 ± 0.058^c^
	SR (%)	90.45 ± 5.71^a^	89.07 ± 6.08^b^	87.61 ± 7.09^c^
	FCR (kg / kg)	2.74 ± 0.19^a^	2.76 ± 0.21^b^	2.78 ± 0.22^b^
Piglets-finishing	FEI (g)	218.30 ± 34.04^a^	211.23 ± 35.08^b^	203.70 ± 36.86^c^

*Exclusion criteria: blank data, ADG ≤ 0 kg, FCR < 1 and ≥ 5, BW of finishing pigs < 65 kg, SR < 65%, and feeding days < 120 d. *ADFI* average daily feed intake, *FEI* fattening efficiency index, *BW* body weight, *ADG* average daily gain, *SR* survival rate, *FCR* feed conversion rate.

The proportions of seven categorical variables with significant difference related to FEI during the piglets-finishing stages in three levels of performing batches were shown in Table 5. The HP batches had a lower proportions in owned source of piglets, number of the purchasing piglets in spring and winter, number of batches in the East and North regions (East China, North China, Northeast and Northwest), and five-way crossbred pigs. On the contrary, they had a higher proportions in the use of CCTV and wastes treatment system.

**Table 5:** Comparisons of the proportions of seven categorical variables of significant correlation with FEI at piglets-finishing stages in high, moderate, and low-performing batches of commercial pigs.

Items		N*	High-performing batches (n = 1,599) (%)	Moderate-performing batches (n = 1,603) (%)	Low-performing batches (n = 1,615) (%)
Source of piglets	Owned	3424	69.04	71.30	72.88
	Internal	1186	26.77	23.96	23.16
	External	207	4.19	4.74	3.96
Season for piglets	Spring	1122	17.13	23.08	29.60
	Summer	1467	29.83	33.37	28.17
	Autumn	1493	40.03	27.64	25.39
	Winter	735	13.01	15.92	16.84
Region	East China	1385	24.27	27.51	34.43
	North China	270	4.00	5.99	6.81
	South China	1415	33.33	29.32	25.51
	Central China	580	14.01	11.92	10.22
	Northwest	444	8.26	11.03	8.36
	Northeast	169	2.37	3.87	4.27
	Southwest	554	13.76	10.36	10.40
Crossbred	Three-way	3545	77.86	72.99	69.97
	Five-way	1185	19.76	25.76	28.24
	Two-way	87	2.38	1.25	1.80
Area of concrete grids	1/3	1655	35.29	40.34	34.80
	2/3	1597	36.47	35.52	34.60
	1/2	1241	28.24	24.14	30.60
CCTV	Yes	4478	98.31	97.15	97.15
	No	113	1.69	2.85	2.85
Wastes treatment system	Yes	4561	99.67	99.47	98.84
	No	31	0.33	0.53	1.16

*Blank data was excluded. *CCTV* closed circuit television video.

In order to find the change of FEI at different stages, the batches of high, medium and low FEI at the finishing stage was tracked back to the same batches at the nursery stage. The same classification was still the most, followed by the adjacent classification (Table 6).

**Table 6:** Changes in the corresponding proportion of FEI for the same batch at nursery and finishing stage.

Classification of batches with different production performance at finishing stage	Proportion (%)	Value range of FEI (g)	Number of batches	Classification of batches with different production performance at nursery stage		
				HP (%)	MP (%)	LP (%)
LP	0–20	121–182	841	23.5	35.2	41.3
MP	45–65	204–223	865	36.2	37.7	26.1
HP	80–100	243–280	847	43.0	33.5	23.5

*FEI* fattening efficiency index, *HP* high-performing batches, *MP* moderate-performing batches, *LP* low-performing batches.

**Fig. 2:**
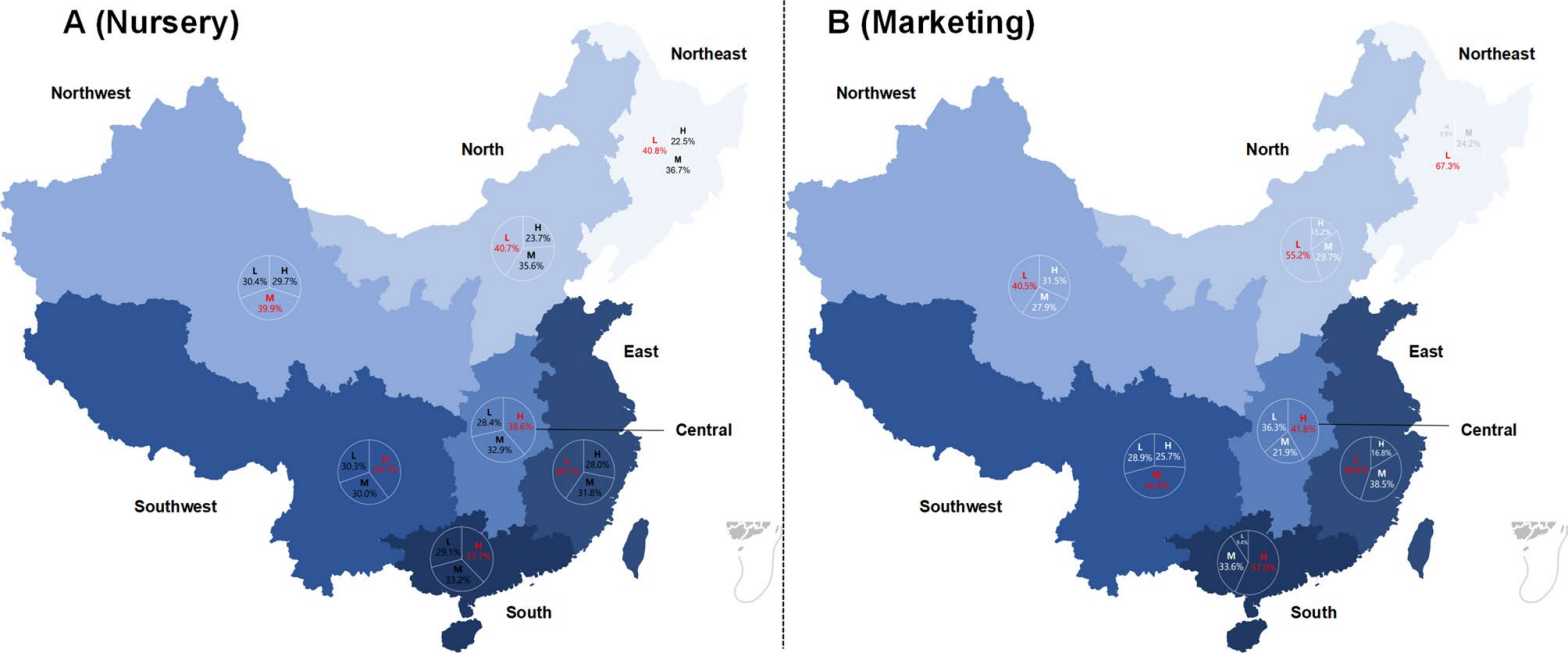
The distribution proportion of the three levels of performing batches in seven major geographical regions of China at nursery stage and finishing stage. The darker the background color, the higher the quantity. At nursery stage, the number of batches in high-performing (HP), moderate-performing (MP) and low-performing (LP) was 1,599, 1,603 and 1,615, respectively. At finishing stage, the number of batches in HP, MP and LP was 1,498, 1,494 and 1,420, respectively. The red font represented the highest proportion. The open-source programming language R version 4.3.0 ( https://mirrors.bfsu.edu.cn/CRAN/) was used to create the map, along with the R package mapdata version 2.3.1 ( https://cran.r-project.org/web/packages/mapdata/index.html) and ggplot2 version 3.5.0 (https://ggplot2.tidyverse.org/). *H* high-performing batches, *M* moderate-performing batches, *L* low-performing batches.

The geographical distribution proportion of the different performing batches was shown in Fig. 2. The regions with the highest proportion of HP batches at nursery stage were located in Southwest, Central China and Southern China, respectively (Fig. 2A). At finishing stage, the regions was reduced to Southern China and Central China, but the proportion of HP batches in Southern China was raised to 57.7% (Fig. 2B).

## Discussion

This research extended the previous study by further refining the application of the FEI^11^. This was achieved by identifying 28 variables that impacted the FE of commercial pigs in large-scale farms through a multivariable linear regression model. Furthermore, this study was the first to compare the FE of pig groups with varying production levels, thereby facilitating the development of more targeted intervention strategies to enhance production performance. As indicated in Table 3, the production stage had a significant impact on FE. Taking the age of 70 days as the cut-off point, we analyzed the effects of the piglets-nursery stage and growing-finishing stage on the FEI of the piglets-finishing stage. Overall, the FEI at nursery stage was higher than the reference, but the FEI at piglets-finishing stage was lower (Table 1). The ADG of the whole stage was lower by 7.14%, the FCR was lower by 6.15%, and the SR was lower by 9.28%. The combined effect led to a 17.39% lower of FEI at piglets-finishing stage.

Under the condition that ADG and SR were lower than the reference, low FCR became a key factor for high FEI at nursery stage. In practice, the company implemented a mode of manual feeding 4–7 times a day during the first month, so the actual feeding amount may be much lower than that of ad libitum feeding. In addition, since the outbreak of ASF in China in August 2018, the production operations in breeding farms have been affected to varying degrees. For example, the restriction of cross-fostering and lactation in batches in farrowing rooms, the large number of gilts in breeding herds, and the lower parity of sows may affect the quality of piglets, thereby affecting production performance of commercial pigs. Although the ADFI was not calculated in the formula of FEI, it had impact on FCR. If disease factors were excluded, the decrease in ADFI may be due to the environment temperature being higher than the thermoneutral zone, which led to reduce the heat production related to digestible and metabolic utilization of feed^12^. However, we were not able to collect information on batch diseases and environmental temperature to determine the reason for the decrease in ADFI. Li et al*.*^13^ reported that the weaning piglets in a group farrowing system spent more time lying down and less time standing and feeding when they were transferred to the fattening farm. These behaviors contributed to retain more energy to gain BW and spend less energy on physical activity, which may help improve ADG and FCR^14^. Early ADG may affect the behavior and performance of growing and finishing stages^15^. Therefore, early feeding and performance evaluation should be paid attention to in production practice.

The FEI at growing-finishing stage was about 15% lower than the reference, and the difference between groups was significantly smaller than that at piglets-nursery stage, indicating compensatory growth in MP and LP batches, but overall growth performance was still low (Table 4). Practically, such results may be influenced by data culling (for accurate calculation of FEI). Compared with other production stages, external environmental factors had a more severe impact on the growing-finishing stage, which may be due to increased sensitivity of pigs to heat and a natural decrease of growth rate at the later stage^16^. Reduction in feed intake caused by any factor will result in a insufficient nutritional intake and a decreased BW gain, hiding the deterioration of FCR, thereby affecting FEI. Notably, the consistency of batches with the same growth performance from the nursery stage to the finishing stage was as low as about 40% (Table 6). This may be influenced by multiple factors. Among the seven categorical variables that significantly affect FEI at piglets-finishing stage, the factors of season, region and crossbred of HP and LP batches differed by more than 5%.

### Season

In general, the optimal production performance of growing-finishing pigs can be supported within an environmental temperature range of 10 °C to 23.9°C^17^. Compared to other animals, pigs were more sensitive to high temperatures due to their higher metabolic heat production, greater fat deposition, and lack of sweat glands^18^. Myer & Bucklin^19^ showed that the feed intake of finishing pigs weighing over 30 kg was lower in summer than in other seasons, which may result in a deficiency of amino acids such as lysine and/or isoleucine^20^. Conversely, in winter, pigs need to increase their feed consumption to maintain normal growth rates^21^. In cold seasons, a minimal ventilation was often used to maximize insulation in pig farms, which led to increased concentrations of air pollutants^22^. This can directly or indirectly impair immune function, induce metabolic changes, trigger respiratory diseases, and decrease the growth performance^23,24^. Therefore, extreme weather conditions in summer and winter were not suitable for the growth of commercial pigs, and it was particularly important to maintain the pig herds in the thermoneutral zone for optimal growth.

During the early weaning stage, wooden boards (in the southern regions), floor heating (in the northern regions), accompanied by heat lamps were used to provide a higher indoor temperature (27 °C-32°C). With the growth of pigs, the use of insulation facilities was gradually reduced. China is located in the northern hemisphere, and the average temperature in Autumn ranges from 10 °C to 22 °C, which coincides with the suitable for growing-finishing pigs. Among the HP batches, 40.03% of piglet introductions occurred in autumn, with the lowest proportion in winter at 13.01%, and their FEI was much higher than that of other production level batches (Table 3), which was reasonable.

### Region

Due to China's vast territory and rich landform, there were obvious differences in road transportation, labor wages, breeding techniques, feed prices, and pork prices in different regions, each with its own advantages and disadvantages. For example, the eastern region had a higher investment intensity in environmental regulation and pollution control compared to the central and western regions. The western region was relatively underdeveloped economically, making it easier to choose the sites suitable for the construction of large-scale pig farms. The central, eastern, and southern regions had dense populations and relatively developed economies, allowing them to engage in technological innovation and improve management^25^. In our study, compared with other production level batches, the HP batches were more distributed in the southern and central regions (Table 5), indicating a regional advantage. Furthermore, the geographical distribution proportions of different production level batches reveal that the proportion of HP batches at nursery stage expanded further in the central and southern regions, while the proportion of LP batches was high in North China, Northeast China, and the eastern region (Fig. 2). This suggested that the central and southern regions had advantages in both internal production efficiency and external economic and demographic impact. It was recommended to focus on these areas and appropriately expand the scale.

### Crossbred

Our study confirmed the benefits of crossbred in enhancing commercial pig performance. The three-way cross (Duroc x Landrace x Yorkshire (DLY)) was prevalent, constituting over 70% of the batches (Table 5), and demonstrated superior growth traits compared to two-way crossbred. The three-way cross, particularly with 75% Hampshire inheritance, excelled in both productive performance and carcass quality, especially lean meat production^26^. This aligned with the widespread adoption of DLY hybridization in China's modern pig farms for improving meat quality and growth efficiency. The three-way and five-way crossbred were optimal for markets that prioritized lean meat, while the two-way crossbreeding was ideally suited for breeding purposes^27^. The crossbred adeptly addressed the varied demands of consumers and accommodated the diverse conditions of production environments. Compared to three-way, five-way crossbred introduced more genetic variations, which may further improve the growth performance and meat quality of pigs.

### Others

From a behavioral perspective, pigs spent about 80% of the day lying, and as their BW increased, they tended to lie down longer^28^. Therefore, the thermal comfort provided by the floor was crucial. Generally, pigs lied on insulated solid floors. When the temperature rose, more pigs tended to lie on slatted floors because the surface temperature of the slatted floor was typically 3 °C to 5 °C lower than that of the insulated solid floor^29^. Therefore, it was essential to match suitable slatted area in different climatic conditions. This study found that at different production levels, the proportion of 2/3 concrete grids of floor was all around 35%. HP pig farms were mainly distributed in the central and southern regions of China (Table 5). This kind of floor was more conducive to cooling, so it may be one of the reasons for their high production performance. In contrast, it may be unsuitable for the northern regions.

The CCTV system was used for 24-h monitoring of image information inside and outside the pig farm. These cameras have recording functions and can be used to find out the reasons for theft or accidents, or to check the status of the pig farm and animals in real time^30^. Although the initial investment in the CCTV system may be high, its long-term benefits in improving pig welfare, reducing disease occurrence, and increasing production efficiency may bring better economic benefits and environmental sustainability^31^. In our study, we found that the use of CCTV was higher in HP batches, confirming this statement. Therefore, future research and practice should further explore the potential of CCTV technology in smart farming systems.

The treatment of waste generated by large-scale pig farms was a challenge. In recent years, the Chinese government has implemented a series of measures to strengthen the pollution control caused by livestock and poultry farming, promote the conversion of animal manure into fertilizer in the field, and facilitate the sustainable development of agriculture^32^. By improving the manure management systems in pig production facilities, significant advantages could be achieved in terms of animal health and productivity^33^. In this study, we found that HP pig farms had the highest utilization rate of waste treatment systems, which can be attributed to the improved environment benefiting animal health and productivity.

### Non-significant factors

While a multitude of factors were assessed to determine their impact on the FE of commercial pigs, some factors, including the stock and gender, did not show statistical significance. This lack of significance could stem from high variability in the data, interactions with other significant factors, a narrow distribution of variables, or limitations in data collection and analysis methods^22^. However, the non-significance did not imply that these factors were unimportant in actual production. In fact, under different production environments or management conditions, they may still play a crucial role. For instance, Agostini et al*.*^22^ found that batches with less than 800 pigs presented (*P* < 0.01) higher ADG. In the production model of Brazilian cooperatives, the gender of pigs significantly affected daily feed intake and FCR^34^. Therefore, future research should further explore the potential roles of these non-significant factors under various production systems and environmental conditions to fully understand all the relevant factors affecting the FE of commercial pigs.

The evidence presented in this study demonstrated that the FEI had the potential to serve as a valuable tool for enhancing the production efficiency of commercial pigs. In order to implement these findings, it was recommended that producers focused on the key variables identified, such as ADG, FCR, SR and feeding days, especially during the early stage of production. It would be beneficial for future research to investigate the applicability of the FEI across diverse production systems, including intensive and extensive farming, as well as in different environmental conditions, such as climate and housing types. A deeper understanding of the interactions between these factors and FEI will facilitate the optimisation of pig production efficiency.

## Conclusions

This study presented a comprehensive framework for the assessment and improvement of FE in commercial pigs, based on the FEI. It was noteworthy that the FEI for commercial pig batches in 2022 was 17.39% lower than the reference value. This decline was influenced by a range of factors, including variations in FEI across different stages, the quality of piglets, the geographical location of farms, production performance during the nursery and finishing stage, the area of concrete grids, and the employment of waste treatment systems and CCTV. In batches with consistent feeding days, HP batches exhibited significantly higher ADFI during the nursery stage, along with better BW, ADG, SR, FCR and FEI at different stages. Furthermore, the presence of a high proportion of autumn-born piglets, three-way crossbred piglets, and farms situated in Southern, Central, and Southwest China may also contribute to high production performance. It was hypothesized that the decline in FE began at the end of the nursery stage and persisted through to the fattening stage. By identifying the key variables and suggesting targeted interventions, producers may enhance their production outcomes. Further research is required to refine these strategies by examining the impact of different production systems and environmental conditions on FEI, thereby facilitating the development of appropriate solutions for diverse farming systems.

### Supplementary Information

Supplementary Information.

## Data Availability

The datasets generated and analyzed during the current study are not publicly available due to the confidentiality of producers, but are available from the corresponding author on reasonable request.

## References

[1] Zhang, Q. F. & Zeng, H. Politically directed accumulation in rural China: The making of the agrarian capitalist class and the new agrarian question of capital. *J. Agrar. Chang.* **21**, 677–701 (2021). 10.1111/joac.12435

[2] Havlíček, J. *et al.* Efficiency of pig production in the Czech Republic and in an international context. *Agriculture* **10**, 597 (2020). 10.3390/agriculture10120597

[3] Opriessnig, T., Giménez-Lirola, L. G. & Halbur, P. G. Polymicrobial respiratory disease in pigs. *Anim. Health Res. Rev.* **12**, 133–148 (2011). 22152290 10.1017/S1466252311000120

[4] Park, C. *et al.* A new modified live porcine reproductive and respiratory syndrome vaccine improves growth performance in pigs under field conditions. *Clin. Vaccine Immunol.* **21**, 1350–1356 (2014). 25056364 10.1128/CVI.00377-14 PMC4178575

[5] Lyoo, K. S., Choi, J. Y., Hahn, T. W., Park, K. T. & Kim, H. K. Effect of vaccination with a modified live porcine reproductive and respiratory syndrome virus vaccine on growth performance in fattening pigs under field conditions. *J. Vet. Med. Sci.* **78**, 1533–1536 (2016). 27264966 10.1292/jvms.16-0137 PMC5059386

[6] Gaillard, C., Brossard, L. & Dourmad, J. Y. Improvement of feed and nutrient efficiency in pig production through precision feeding. *Anim. Feed Sci. Technol.* **268**, 114611 (2020). 10.1016/j.anifeedsci.2020.114611

[7] Kerr, B. J., Yen, J. T., Nienaber, J. A. & Easter, R. A. Influences of dietary protein level, amino acid supplementation and environment temperature on performance, body composition, organ weights and total heat production of growing pigs. *J. Anim. Sci.* **81**, 1998–2007 (2003). 12926782 10.2527/2003.8181998x

[8] Homma, C. *et al.* Estimation of genetic parameter for feed efficiency and resilience traits in three pig breeds. *Animal.* **15**, 100384 (2021). 34757251 10.1016/j.animal.2021.100384

[9] Remus, A., Hauschild, L., Létourneau-Montminy, M. P. & Pomar, C. Estimating amino acid requirements in real-time for precision-fed pigs: The challenge of variability among individuals. *Animals* **11**, 3354 (2021). 34944131 10.3390/ani11123354 PMC8698096

[10] Eriksen, E. Ø., Nielsen, J. P., Agerlin, M. V., Christensen, A. E. & Pedersen, K. S. Easy and reliable assessment of the prevalence of porcine post-weaning diarrhoea. *Prev. Vet. Med.* **220**, 106041 (2023). 37866129 10.1016/j.prevetmed.2023.106041

[11] Guan, R., Wu, J., Wang, Y., Cai, Q. & Li, X. Comparative analysis of production performance and fattening efficiency of commercial pigs in China for two consecutive years. *Sci. Rep.* **13**, 8154 (2023). 37208541 10.1038/s41598-023-35430-y PMC10199096

[12] Oladele, P. *et al.* Effect of a carbohydrase admixture in growing pigs fed wheat-based diets in thermoneutral and heat stress conditions. *J. Anim. Sci.* **99**, skab254 (2021). 34460910 10.1093/jas/skab254 PMC8562353

[13] Li, Y. Z., Wang, L. H. & Johnston, L. J. Effects of farrowing system on behavior and growth performance of growing-finishing pigs. *J. Anim. Sci.* **90**, 1008–1014 (2012). 22021805 10.2527/jas.2011-4050

[14] Fornós, M. *et al.* The feeding behaviour habits of growing-finishing pigs and its effects on growth performance and carcass quality: A review. *Animals* **12**, 1128 (2022). 35565555 10.3390/ani12091128 PMC9099574

[15] Quiniou, N., Renaudeau, D., Collin, A. & Noblet, J. Effets de l’exposition au chaud sur les caractéristiques de la prise alimentaire du porc à différents stades phydiologiques. *INRAE Prod. Anim.* **13**, 233–245 (2000). 10.20870/productions-animales.2000.13.4.3783

[16] Hörtenhuber, S. J. *et al.* The effect of climate change-induced temperature increase on performance and environmental impact of intensive pig production systems. *Sustainability* **12**, 9442 (2020). 10.3390/su12229442

[17] Mun, H. S., Rathnayake, D., Dilawar, M. A., Jeong, M. G. & Yang, C. J. Effect of ambient temperature on growth performances, carcass traits and meat quality of pigs. *J. Appl. Anim. Res.* **50**, 103–108 (2022). 10.1080/09712119.2022.2032084

[18] Gourdine, J. L., Rauw, W. M., Gilbert, H. & Poullet, N. The genetics of thermoregulation in pigs: a review. *Front. Vet. Sci.* **8**, 770480 (2021). 34966808 10.3389/fvets.2021.770480 PMC8711629

[19] Myer, R. O. & Bucklin, R. A. Effect of season (summer vs. fall) and diet nutrient density on performance and carcass characteristics of growing-finishing swine. *Trans. ASAE* **45**, 807–811 (2002).

[20] Noblet, J., Le Dividich, J. & van Milgen, J. Thermal environment and swine nutrition. In *Swine Nutrition.* (eds. Lewis, A. J. & Southern, L. L.) 539–564 (CRC Press, 2001).

[21] Rauw, W. M. *et al.* Impact of environmental temperature on production traits in pigs. *Sci. Rep.* **10**, 2106 (2020). 32034216 10.1038/s41598-020-58981-w PMC7005870

[22] Agostini, P. S. *et al.* Descriptive study of production factors affecting performance traits in growing-finishing pgis in Spain. *Span. J. Agric. Res.* **11**, 371–381 (2013). 10.5424/sjar/2013112-3011

[23] Pluske, J. R., Kim, J. C. & Black, J. L. Manipulating the immune system for pigs to optimise performance. *Anim. Prod. Sci.* **58**, 666–680 (2018). 10.1071/AN17598

[24] Buoio, E., Cialini, C. & Costa, A. Air quality assessment in pig farming: The Italian classyfarm. *Animals* **13**, 2297 (2023). 37508074 10.3390/ani13142297 PMC10376095

[25] Zhong, S., Li, J. & Zhang, D. Measurement of green total factor productivity on Chinese pig breeding: From the perspective of regional differences. *Environ. Sci. Pollut. Res.* **29**, 27479–27495 (2022). 10.1007/s11356-021-17908-2 34982382

[26] Kadirvel, G. *et al.* Productive and reproductive performances of two-breed and three-breed pig crosses with Niang Megha, Hampshire and Duroc inheritance reared under subtropical Eastern Himalayan hilly climate. *Trop. Anim. Health Prod.* **53**, 1–9 (2021). 10.1007/s11250-020-02474-5 33409648

[27] Lei, H. G. *et al.* Comparison of the meat quality, post-mortem muscle energy metabolism, and the expression of glycogen synthesis-related genes in three pig crossbreeds. *Anim. Prod. Sci.* **55**, 501–507 (2014). 10.1071/AN13484

[28] Ekkel, E. D., Spoolder, H. A. M., Hulsegge, I. & Hopster, H. Lying characteristics as determinants for space requirements in pigs. *Appl. Anim. Behav. Sci.* **80**, 19–30 (2003). 10.1016/S0168-1591(02)00154-5

[29] Huynh, T. T. T., Aarnink, A. J. A., Spoolder, H. A. M., Verstegen, M. W. A. & Kemp, B. Effects of floor cooling during high ambient temperatures on the lying behaviour and productivity of growing finishing pigs. *Trans. ASAE* **47**, 1773–1782 (2004). 10.13031/2013.17620

[30] Hwang, J. & Yoe, H. Study of the ubiquitous hog farm system using wireless sensor networks for environmental monitoring and facilities control. *Sensors* **10**, 10752–10777 (2010). 22163497 10.3390/s101210752 PMC3231097

[31] Racewicz, P. *et al.* Welfare health and productivity in commercial pig herds. *Animals* **11**, 1176 (2021). 33924224 10.3390/ani11041176 PMC8074599

[32] Li, J. *et al.* Development and environmental impacts of China’s livestock and poultry breeding. *J. Clean. Prod.* **371**, 133586 (2022). 10.1016/j.jclepro.2022.133586

[33] Khoshnevisan, B. *et al.* A critical review on livestock manure biorefinery technologies: Sustainability, challenges, and future perspectives. *Renew. Sust. Energ. Rev.* **135**, 110033 (2021). 10.1016/j.rser.2020.110033

[34] Silva, C. A. *et al.* Characterization and influence of production factors on growing and finishing pig farms in Brazilian cooperatives. *Rev. Bra. Zootecn.* **46**, 264–272 (2017). 10.1590/s1806-92902017000300012

