# Supplementary material for: Identification of factors affecting fattening efficiency of commercial pig herds and analysis of their impact at different performance levels
Source: Sci Rep. 2024 Aug 29;14:20105. doi: 10.1038/s41598-024-70900-x (PMC11362584; doi:10.1038/s41598-024-70900-x)
Supplement: Supplementary file 1 — Supplementary Information. [file 41598_2024_70900_MOESM1_ESM.docx]

**Table S1** The descriptions of features of batch data.

| **Features** | **Descriptions / Formulas** |
| --- | --- |
| **Farm facilities** |  |
| Area of concrete grids | The area of concrete grids includes 1/3, 2/3, and 1/2, with the remainder being solid floor. |
| Enclosure | The pig farm is enclosed by a solid fence. |
| Bio-safety zoning | Zones of bio-security management: the periphery, productive area, living area and fecal sewage treatment area were separated. |
| Disinfection channel | There is a dedicated disinfection channel for personnel and goods. |
| Wastes treatment system | The farm has a manure solid-liquid separation system and is equipped with wastewater treatment equipment. |
| Automatic feeding system | The farm owns feeding towers, and automatic feeding  lines can meet the maximum feed intake for pigs over a seven-day period. |
| Environmental control system | Automatic environmental control system and mechanical ventilation system (climate controller for controlling fans of different sizes). |
| Bio-safety disposal | The harmless treatment facilities for dead pigs. |
| CCTV | CCTV cameras are installed around the perimeter of the pig farm and inside the pig houses. |
| **General information of piglets** | |
| Introduced date | The date of introducing piglets. |
| Company | The company managing the pig farm. |
| Batch number | Batch number. |
| Source farm | Piglet source farm. |
| Breed | Crossbred, eg. three-way, five-way and two-way. |
| Source of piglets | Piglets from the same company, piglets from internal company, and piglets from external company. |
| Gender | Mixed, barrow and female. |
| Number of piglets introduced | The number of piglets introduced in a batch. |
| BW of piglets (kg) | The average BW of piglets in a batch. |
| Total BW introduced (kg) | The total BW of piglets in a batch. |
| Initial age (d) | The average age of piglets in a batch. |
| **Production performance at nursery stage** | |
| Days during the piglet-nursery stage (d) | = 70 days - Initial days. |
| BW of nursery pigs at 70 days (kg) | Used to calculate the average BW gain during the piglet-nursery stage. |
| SR of nursery pigs at 70 days (%) | Used to calculate the FEI during the piglet-nursery stage. |
| Gain BW during the nursery stage (kg) | = BW of nursery pigs at 70 days - BW of piglets. |
| ADG during the nursery stage (kg) | = Gain BW during the nursery stage / Days during the piglet-nursery stage. |
| Feed consumption during the nursery stage (kg) | Used to calculate the FCR during the nursery stage. |
| ADFI during the nursery stage (kg) | = Feed consumption during the nursery stage / The number of piglets introduced / Days during the piglet-nursery stage. |
| FCR during the nursery stage (kg / kg) | = Feed consumption during the nursery stage / Gain BW during the nursery stage. |
| FEI during the nursery stage (g) | = SR of nursery pigs at 70 days (%) × ADG during the nursery stage (kg) × 1000 / FCR during the nursery stage. |
| **Production performance at nursery stage during piglets-finishing stage** | |
| Feeding days (d) | Days during the piglet-finishing stage. |
| Number of finishing pigs (n) | Used to calculate the SR during the piglet-finishing stage. |
| Number of finishing pigs during the piglets-finishing stage (n) | The final number of live pigs marketed in a batch. |
| BW of finishing pigs during the piglets-finishing stage (kg) | The final BW of live pigs marketed in a batch. |
| ADG during the piglets-finishing stage (kg) | = Gain BW during the piglet-finishing stage / Days during the piglet-finishing stage. |
| SR during the piglets-finishing stage (%) | Number of finishing pigs during the piglets-finishing stage / Number of piglets introduced. |
| Feed consumption during the piglets-finishing stage (kg) | Used to calculate the FCR during the piglets-finishing stage. |
| FCR during the piglets-finishing stage (kg / kg) | = Feed consumption during the piglets-finishing stage / Gain BW during the piglets-finishing stage. |
| FEI during the piglets-finishing stage (g) | = SR of finishing pigs (%) × ADG during the piglets-finishing stage (kg) × 1000 / FCR during the piglets-finishing stage. |

*CCTV* closed circuit television video, *BW* body weight. *SR* survival rate, *ADG* average daily gain, *ADFI* average daily feed intake, *FCR* feed conversion rate, *FEI* fattening efficiency index.
